# Obstetric healthcare experiences and information needs of Dutch women in relation to their vegan diet during pregnancy

**DOI:** 10.1016/j.pmedr.2024.102916

**Published:** 2024-10-24

**Authors:** Deidre Meulenbroeks, Daisy Jonkers, Hubertina Scheepers, Jessica Gubbels

**Affiliations:** a Maastricht University Medical Centre, Department of Obstetrics & Gynaecology, GROW – Research Institute of Oncology and Reproduction, P. Debyelaan 25, 6202 AZ Maastricht, the Netherlands; b Maastricht University, NUTRIM Institute of Nutrition and Translational Research in Metabolism, Department of Internal Medicine, 6200 MD Maastricht, the Netherlands; c Maastricht University, NUTRIM, Institute of Nutrition and Translational Research in Metabolism, Department of Health Promotion, 6200 MD Maastricht, the Netherlands

**Keywords:** Plant-based diet, Vegan, Diet, Vegetarian, Pregnancy, Obstetric healthcare, Midwifery

## Abstract

**Objectives:**

The prevalence of a vegan (strict plant-based) diet is increasing. Adequate access to nutritional information for pregnant women on a vegan diet is vital, as optimal maternal nutrition is important in order to prevent maternal and fetal complications. This study evaluated the obstetric healthcare experiences and information needs of pregnant women following a vegan diet in the Netherlands.

**Methods:**

An online survey examined women’s experiences with obstetric healthcare in relation to their vegan diet during pregnancy. In total, 195 women, all of whom followed a vegan diet during their pregnancy and gave birth between January 2018 and April 2020, completed an online survey about their experiences. Descriptive statistics were used to present the data.

**Results:**

In total, 36.5% of participants reported that an obstetric healthcare provider had inquired about their diet in general during the first prenatal consultation, and an additional 43.1% of participants proactively informed their healthcare provider about their diet. Only 19.0% of all participants received specific information about their vegan diet during pregnancy, and 25.2% reported having had extra checks, mostly blood tests, during their pregnancy. Of all participants, 35.9% would have preferred to receive additional information regarding their diet.

**Conclusions:**

Our data shows that only a minority of pregnant women following a vegan diet received information and recommendations about their diet from their healthcare providers. It is our recommendation that healthcare providers inquire about pregnant women’s diets, and refer those following a vegan diet to reliable sources of dietary information or to a specialized dietitian.

## Highlights

• In their first antenatal consult, 36.5% of pregnant women were asked about diet.
• A minority of pregnant vegan women received recommendations concerning their diet.
• Vegan nutritional education among obstetrical healthcare providers is needed.
• Awareness among obstetricians of the importance of nutrition in pregnancy needs to be improved.

## 1 Introduction

Worldwide, the popularity of a vegan diet is increasing (The Vegan Society, 2024). This diet excludes all animal products, such as meat and fish, dairy, eggs, and honey (The Vegan Society, 2024). People often choose this diet because of its potential health benefits, environmental or ethical concerns, and/or as part of the vegan lifestyle, which seeks to exclude all forms of exploitation of, and cruelty to, animals for food, clothing, or any other purpose (Melina et al., 2016). In the Netherlands, the prevalence of adopting a vegan lifestyle has risen to 0.4 % in 2023, but estimations range up to 1.5 % depending on the methods used to assess prevalence. (Nederlandse Vereniging voor Veganisme, 2024). Worldwide, 3 % of the population has a vegan lifestyle, according to a study that collected data from 28 countries (Nederlandse Vereniging voor Veganisme, 2024). The majority of vegans are women in the fertile age range (Allès et al., 2017, Coppola, 2019). This suggests that the number of pregnant women following a vegan diet during pregnancy is also increasing, although prevalence data for this specific group are lacking.

Maternal prenatal nutrition affects maternal health and is also crucial for the (unborn) child, affecting fetal neurodevelopment and potentially having lifelong mental health consequences (Schwarzenberg et al., 2018). Inadequate maternal prenatal nutrition is associated with a higher incidence of metabolic syndrome, diabetes, and cardiovascular disease later in the child's life (Lowensohn et al., 2016). Avoiding severe nutrient deficiencies by making improvements in women's nutrition both before and during pregnancy contributes to optimal fetal growth, favorable obstetrical outcomes, improved perinatal survival, and the potential for better long-term health in both the mother and offspring (Marshall et al., 2022).

International recommendations about vegan diets during pregnancy are scarce and inconsistent. For instance, the German Nutrition Society indicates that they cannot make a clear recommendation for or against a vegan diet during pregnancy and lactation, due to the limited available data. (Klug et al., 2024). The American Academy of Nutrition and Dietetics states that a well-planned vegan diet can be appropriate, satisfy nutrient needs, and promote normal growth at all life cycle stages, including pregnancy and lactation (Melina et al., 2016). The Dutch Nutrition Centre recommends that pregnant women with a vegan diet consult a dietitian (Dutch Nutrition Centre (Voedingscentrum), 2024). In the Netherlands, up to 3 h per year with a dietitian is paid for by the mandatory standard health insurance (after paying the deductible excess). Additionally, the Dutch Organization of Midwifery advises vegan pregnant women to add omega-3 and vitamin B12 supplements to their diet, and, in case of insufficient intake, also iodine, calcium, and zinc supplements, in the appropriate dosage (KNOV, 2024).

One Danish study measured the nutritional intake of 18 vegan participants between 1996 and 2002, and reported that pregnant women following a vegan diet had lower protein, retinol, vitamin B12, vitamin D, calcium, and iodine intakes compared to omnivorous women (Hedegaard et al., 2024). However, when the women’s dietary supplement intake was added to the dietary contribution, only the median intake of vitamin D remained below the recommended levels (Hedegaard et al., 2024). Vitamin D deficiency is common among all pregnant women; therefore, vitamin D supplements are recommended regardless of maternal dietary choices (Saraf et al., 2016). In another study conducted in Israel, the maternal and umbilical cord blood of women following a vegan diet showed no significant difference in measurements of hemoglobin, ferritin, vitamin B12, folic acid, and albumin compared to women on an omnivorous diet (Avnon et al., 2020). There have been a small number of studies, with limited sample sizes resulting in insufficient statistical power, that have investigated pregnancy outcomes of women following a vegan diet. Some studies performed in Israel and Italy have reported an association between a vegan diet during pregnancy and an increased risk of having a child with a significantly lower birth weight (Avnon et al., 2020, Kesary et al., 2020, Ferrara et al., 2019). Other studies have shown the potentially protective effect of a maternal vegan diet against excessive maternal weight gain (Avnon et al., 2020, Kesary et al., 2020). Healthy weight gain in pregnancy has a potentially protective effect against gestational diabetes and hypertensive disorders (Champion et al., 2020). These preliminary findings show the importance of adequate nutritional information for pregnant women following a vegan diet. Further insight is needed into how much information and advice – if any – pregnant women are provided with. This is of vital importance, considering the recommendation that vegan women should be receiving vegan-specific dietary advice (Agnoli et al., 2017).

In line with this, the aim of our study was to evaluate the obstetric healthcare experiences and information needs of women residing in the Netherlands in relation to their vegan diet during pregnancy.

## 2 Methods

A cross-sectional retrospective survey-based study with a nonrandom convenience sample was conducted between April and May 2020. The EQUATOR network’s CROSS and CHERRIES Checklist was used to report this online survey study (Supplement 1). This study was conducted according to the guidelines laid down in the Declaration of Helsinki. All procedures involving research study participants were approved by the Ethics Review Committee of the Faculty of Health Medicine and Life Sciences of Maastricht University as part of the license of the Master Health Education and Promotion program, registration code FHML/HEP_202.074.

### 2.1 Participants and procedures

Women residing in the Netherlands who were following a vegan diet during pregnancy and who gave birth between January 2018 and April 2020 were eligible for inclusion in the study. A vegan diet was defined as a plant-based diet with ingestion of animal products less than once a month, which is in line with previous definitions (Kesary et al., 2020). Women were excluded if they did not complete the questions regarding the inclusion criteria. No additional inclusion or exclusion criteria were applied. Participants were recruited via newsletters and social media platforms of the Dutch Society of Veganism and ProVeg (an international food awareness organization focusing on plant-based food). Additionally, a call to participate in the study was posted on various social media platforms focusing following a vegan diet and parenthood. At the start of the survey, potential participants were informed about the purpose of the study, the topics in the survey, the required time investment, the data storage, and who the principal investigator was. Subsequently, they were asked to provide informed consent online prior to data collection. Participants did not receive any incentives for completing the survey.

### 2.2 Survey

The online survey was conducted using the survey software Qualtrics, version April 2020 (Qualtrics, Provo, Utah, USA) (Supplement 2). The survey was developed based on the outcomes of preliminary focus groups conducted with 20 women following a vegan diet who were either pregnant, had recently given birth, or had a wish to become pregnant soon (results not published), as well as relevant obstetrical literature (NVOG, 2024, Meulenbroeks et al., 2021). The items used in the current study were part of a longer questionnaire, which includes other topics concerning a vegan diet in pregnancy that do not fall within the scope of this article.

The online survey was pretested by three pregnant women to ensure comprehensibility, resulting in minor adaptations. The survey comprised 31 questions covering various topics and took approximately 10 min to complete (Supplement 2). To check the uniqueness of the participants, they were asked to report their date of birth. In the survey, after being provided with the definition of a vegan diet, women were asked about their diet (‘Were you following a vegan diet during the entire pregnancy, with ingestion of an animal product less than once a month?’), age, highest level of completed education (primary school, secondary school, intermediate vocational education, higher vocational education, university), height (cm) and weight (kg) before pregnancy, and smoking habits during pregnancy (yes/no). Subsequently, participants were asked if they had been to an appointment for their first pregnancy consultation with a midwife or an obstetrician. In the Netherlands, a midwife most often provides low-risk obstetric care (primary care). Women with high-risk pregnancies are cared for by obstetricians in general (secondary care) or academic hospitals (tertiary care) (van der Lee et al., 2014). Additionally, we inquired about any complications they may have had during pregnancy, such as gestational diabetes, hypertension, pre-eclampsia, fetal growth restriction, macrosomia, or other complications. Lastly, questions were asked about their breastfeeding behavior.

The remainder of the questionnaire consisted of questions about the obstetric healthcare experiences and information needs of pregnant women regarding their diet. An example is, ‘Did you have any specific questions or information needs about your vegan diet during your pregnancy?’ (No; Yes, about nutritional intake; Yes, about supplement use; Yes, about the effect of my diet on the growth of the baby; Yes, about potential maternal complications during pregnancy; Yes, about potential fetal complications during pregnancy; Yes, about nutritional intake during lactation; Yes, about supplements use during lactation; Yes, other, namely …; multiple answers possible). Other examples of questions are ‘Did your midwife or obstetrician ask you about your diet? and ‘Do you think your obstetric healthcare provider should have asked you about your diet?’ All questions in the survey had a non-response option such as ‘not applicable’ or ‘I don’t know’. Respondents could review and change their answers before sending the survey in.

### 2.3 Data processing and statistical analyses

Multiple choice questions were analyzed using descriptive statistics. When participants chose the ‘other, namely….…’ option in multiple choice questions, the answers were analyzed, and more frequently mentioned answers were combined into additional categories; less frequent answers were combined as ‘other’. Results are displayed as frequencies and percentages (categorical data) or as a mean with standard deviation (SD) for parametric continuous data. The survey results were exported from Qualtrics into IBM SPSS Statistics 25.0.

## 3 Results

A total of 257 women participated in the survey. Of these, 48 women were excluded for one of the following reasons: because their diet during pregnancy was not vegan, because they gave birth before January 2018, or because they were still pregnant at the moment of survey completion. Another 14 women were eligible but were excluded because they did not complete all of the inclusion questions. This resulted in a final study sample of 195 women (completion rate 93.3 %).

General information about the included participants can be found in Table 1. Of all participants, 73.3 % had a high educational level, 71.8 % had a normal weight status, and 83.1 % had received their first pregnancy consultation by a midwife. The length of time that participants had been following a vegan diet ranged between 1 and 25 years (median of 4 years). Of all participants, 94.9 % (n = 185) had a partner, of whom 55.4 % (n = 108) were also following a vegan diet. A total of 76.0 % of participants reported an uncomplicated pregnancy (no gestational diabetes, hypertension, pre-eclampsia, fetal growth restriction, or macrosomia), and all children were born alive (Supplement 3. Maternal and fetal outcomes of pregnant women following a vegan diet). Postpartum, 98.5 % (n = 192) started with breastfeeding and 55.9 % (n = 86) of participants did so for at least 6 months (excluding those whose infant was not 6 months old yet).

**Table 1:** Descriptive characteristics of participating women on a vegan diet during pregnancy and who gave birth between 2018 and 2020, residing in the Netherlands (n = 195).

**Characteristics**	***n (%)***^a^
**Age, mean (SD**^b^**)** (*n* = 193)	31 (4.1)

**BMI**^c^**(weight status before pregnancy)**	
Underweight (≤18.49)	8 (4.1)
Normal (18.50–24.99)	140 (71.8)
Overweight (25.00 – 29.99)	30 (15.4)
Obese (≥30.00)	17 (8.7)

**Highest level of completed education**	
Primary school	1 (0.5)
Secondary school	10 (5.2)
Intermediate vocational education	41 (21.0)
Higher vocational education	81 (41.5)
University	62 (31.8)

**Smoking during pregnancy**	
Yes	1 (0.5)
No	194 (99.5)

**Parity**^d^	
1	140 (71.8)
2	39 (20.0)
3	14 (7.2)
4	2 (1.0)

**Primary obstetrical healthcare provider**	
Midwife	162 (83.1)
Obstetrician	33 (16.9)

a *n* (%) = number of participants (percentage).

b SD=standard deviation

c BMI=body mass index in kg/m^2^.

d Parity = the number of births of a fetus with a gestational age of 24 weeks or more, regardless of whether the child was born alive or was stillborn.

The participants' experiences of and need for receiving information about a vegan diet during pregnancy are summarized in Table 2, Table 3, respectively. For the participants who received obstetric care from a midwife (primary care), their midwives inquired about diet in 42.6 % (n = 69) of the participants’ first antenatal consultations. For obstetricians (secondary or tertiary care), this was the case in 18.2 % (n = 6) of the participants’ first antenatal consultations. An additional 43.1 % of participants (n = 66) receiving primary care and 54.5 % (n = 18) of those receiving secondary/tertiary care proactively informed their obstetric healthcare provider about their diet without being asked by the healthcare provider. Of all participants, 19.0 % received recommendations about their diet during pregnancy from their obstetrical healthcare provider. About a quarter of participants (25.2 %) reported receiving extra checks during pregnancy in relation to their diet, which most often included blood tests (e.g*.,* for hemoglobin, vitamin D, and vitamin B12 levels).

**Table 2:** Descriptive results of the experiences of women residing in the Netherlands in relation to receiving information about their vegan diet during pregnancy and who gave birth between 2018–2020 (n = 195).

**Experiences**	***n (%)***^a^
**Obstetrical healthcare provider inquired about diet at first consultation**	
Yes	75 (38.6)
No, but I told him/her about it anyway	84 (43.1)
No, and I did not tell him/her about it	17 (8.7)
I can’t remember	19 (9.7)

**Received information about following a vegan diet during pregnancy from a midwife or gynecologist (n = 178)**	
Yes	37 (20.8)
No	141 (79.2)

**Extra checks because of vegan diet (n = 178)**	
Yes, extra blood check	50 (28.1)
Yes, hemoglobin	34 (19.1)
Yes, vitamins	37 (20.8)
Yes, thyroid function	1 (0.6)
Yes, but I don’t know what checks	2 (1.1)
Yes, extra ultrasound	0 (0.0)
No	123 (69.1)
Do not remember	5 (2.8)

**Received advice to visit a dietitian (n = 178)**	
Yes, but I had already been	4 (2.1)
Yes, and I did go	4 (2.1)
Yes, but I did not go	10 (5.6)
No, and I did not go	151 (77.4)
No, but I did go	9 (4.6)

Type of dietitian (n = 17)*	
Regular dietitian	1 (5.9)
Specialized in pregnancy	3 (17.6)
Specialized in vegan diets	13 (76.5)

**Obstetrical healthcare provider discussed diet at first consultation**	
Yes	75 (38.6)
No, but I told it him/her about it anyway.	84 (43.1)
No, and I did not tell him/her about it.	17 (8.7)
I can’t remember	19 (9.7)

* Participants with prenatal care with the midwife compared to the gynecologist.

a *n* (%) = number of participants (valid percentage, i.e. missing data are excluded).

**Table 3:** Descriptive results of the information needs of women residing in the Netherlands in relation to their vegan diet during pregnancy and who gave birth between 2018 and 2020 (n = 195).

**Information needs**	***n (%)***^a^
**Preferred to have received additional information**	
Yes, on nutritional intake in pregnancy	58 (29.7)
Yes, on supplements during pregnancy	57 (29.2)
Yes, on nutritional intake during lactation	46 (23.6)
Yes, on supplements during lactation	44 (22.6)
Yes, on growth of baby	23 (11.8)
Yes, on fetal complications	13 (6.7)
Yes, on maternal complications	9 (4.6)
Yes, on complications during delivery	4 (2.1)
No	125 (64.1)

**Preference for provider of information**	
The midwife or gynecologist	103 (52.8)
Website	82 (42.1)
I preferred to look for information myself	71 (36.4)
Dietitian	57 (29.2)
Leaflet	48 (24.6)
Nutritional Centre	38 (19.5)
I did not want to receive any extra info	21 (10.8)
General practitioner	11 (5.6)
From someone else	7 (3.6)

a *n* (%) = number of participants (valid percentage i.e. missing data are excluded).

A total of 35.9 % of participants reported having experienced a need for information from their healthcare provider about nutritional intake, supplement use, and the potential risk for maternal and/or fetal complications related to being following a vegan diet during pregnancy or lactation. The preferred timing of receiving information about these topics was preconceptionally (42.9 %), the first trimester (75.7 %), the second trimester (47.1 %), the third trimester (27.1 %), or postpartum (47.1 %). Some participants (n = 3) reported that the expected lack of knowledge about a vegan diet among obstetric healthcare providers was an important reason behind their preference not to receive any additional information and recommendations during their pregnancy check-ups.

In total, 52.8 % of participants indicated that they felt that the responsibility of providing pregnant women with relevant dietary information related to a vegan diet should lie with the midwife or obstetrician, while 29.2 % thought that this was the responsibility of a dietitian. Participants mainly preferred to get this information from a reliable website (42.1 %) or in an informative leaflet (24.6 %). Of the 9.3 % of participants who were advised by their midwife or obstetrician to visit a dietitian, 70.4 % decided not to follow up on this because they felt that they had enough knowledge about their diet themselves or because they felt that a dietitian would not have enough knowledge to provide them with additional recommendations.

## 4 Discussion

This cross-sectional retrospective study evaluated the obstetrical healthcare experiences and information needs of women residing in the Netherlands following a vegan diet during pregnancy. It showed that only a minority of participants received information and recommendations from their obstetrical healthcare providers concerning their diet during pregnancy.

The lack of nutritional guidance during pregnancy is remarkable, as it is widely recognized that maternal prenatal nutrition is important for both the maternal and the (unborn) child’s lifelong health (Schwarzenberg et al., 2018, Marshall et al., 2022). Our study highlights that further research in this field is needed. It is also worth noting that any further research should take into account recent developments in vegan nutrition. To date, the only published study on vegan nutritional intake in pregnancy discusses the nutritional intake from 1996 to 2002. However, the availability of fortified vegan products and the use of supplements has changed significantly in recent years (Hedegaard et al., 2024).

A key challenge for antenatal care clinicians is their inadequate knowledge of nutrition (Lee et al., 2018). Limited time in the curriculums of both medicine and midwifery is spent on nutritional education in general with specific diets such as vegan diets receiving little attention (Arrish et al., 2014, Broad and Wallace, 2018, van Dam-Nolen, 2024). A study among medical students showed that barriers to achieving adequate nutritional education in medical school include the non-prioritization of nutrition education and poor collaboration between the medical faculty and nutrition professionals (Ebinghaus et al., 2024). In line with this, previous research has highlighted a self-reported lack of knowledge about following a vegan diet during pregnancy among obstetric healthcare providers and dietitians in the Netherlands (Meulenbroeks et al., 2021). Correspondingly, obstetric healthcare providers themselves have emphasized the need for improved professional education on nutrition in pregnancy (Meulenbroeks et al., 2021, Ebinghaus et al., 2024). It is clear, then, that better nutritional education among obstetrical healthcare providers is needed, and that awareness about the importance of nutrition in the preconception period, pregnancy, and postpartum needs to be improved. An additional challenge is the lack of clear recommendations available for healthcare workers to use. This is mainly due to the scarcity of studies investigating pregnant women following a vegan diet. However, some recommendations on nutritional intake and supplement use for pregnant women following a vegan diet have been published and can be implemented in daily practice (KNOV, 2024, Agnoli et al., 2017, Koeder and Perez-Cueto, 2024, Baroni et al., 2018).

The effect of dietetic consultation in pregnancy among women following a vegan diet has not yet been studied. However, dietary counseling (sometimes in combination with other lifestyle interventions) during pregnancy has been shown to be effective in terms of various outcomes, including optimizing gestational weight gain management (Mitchell et al., 2017, Buckingham-Schutt et al., 2019, Korpi-Hyövälti et al., 2012), improving the quality of dietary fat intake and increasing the birth weight of infants of pregnant women at high risk of gestational diabetes without an effect on macrosomia (Korpi-Hyövälti et al., 2012), improving maternal glycemic outcomes and the need for medication treatment in women with gestational diabetes mellitus (Koeder and Perez-Cueto, 2024), and preventing iron-deficiency anemia in pregnant women (Skolmowska et al., 2022). Effective dietary counseling is achieved by connecting with and acting upon a client’s motivation and knowledge levels, involving clients and maintaining their involvement, tailoring the modality of the counseling, and developing dietitians' professionalism (Barkmeijer et al., 2022).

Even though pregnant women are often more aware of their nutritional intake than women who are not pregnant or trying to conceive (Szwajcer et al., 2012), only one-third of the participants reported needing nutritional information preconceptionally or during pregnancy. It is possible that some participants were unaware of the influence of potentially harmful nutritional deficiencies on the unborn child (Schwarzenberg et al., 2018). Another explanation for the relatively low percentage of women indicating a need for nutritional information was the lack of knowledge about a vegan diet that they expected from their obstetrical healthcare providers. These results align with the results from a preliminary small sampled focus group study among twenty-five pregnant women (including two vegan women) living in Germany, in which the participants perceived the guidance they received on nutrition during pregnancy as insufficient (Ebinghaus et al., 2024). They reported needing individual nutrition information provided throughout pregnancy, especially in order to reduce uncertainties exacerbated by the internet and social media (Ebinghaus et al., 2024). However, ensuring that these women are able to access this type of information is not easy. A prominent barrier to information access for pregnant women in general is the lack of adequate information resources, as they often receive limited information from their care providers (Meulenbroeks et al., 2021, Ghiasi, 2019).

Our study had several strengths and some limitations that need to be considered. To our knowledge, this is the first study to assess obstetrical healthcare experiences regarding nutritional advice for women following a vegan diet during pregnancy. A relatively large sample of vegan women (n = 195) participated in the current study. However, participants were primarily recruited via (vegan-oriented) social media and might not have been a representative sample of the intended population, resulting in selection bias. Additionally, a general limitation of the online survey design is the lack of control over who is completing the survey and the validity of the answers. There was no registration of the view rate of the questionnaire. There were no cookies used, IP checks or log file analysis performed, or registration prior to joining the study, to indicate the uniqueness of the user. Additionally, there was no check on questionnaires submitted with an atypical timestamp. The respondents of the present study were more often highly educated (73.3 %) compared to the general Dutch population (33 %) (Onderwijsincijfers, 2024). However, as people with a higher educational level more frequently adopt a vegan diet (Lea et al., 2006), the educational level of participants in the present study might be a more or less fair reflection of the population following a vegan diet (Fuschlberger and Putz, 2023). The questionnaire used in the current study was a non-validated self-developed questionnaire, although it was based on results from focus groups and relevant literature in the field (NVOG, 2024, Meulenbroeks et al., 2021). The questions were not randomized or alternated; this could have introduced question order bias. The participants’ medical history, ethnicity, and pregnancy outcomes could potentially influence the findings but were not assessed in the current study. Additionally, some more in-depth questions in the questionnaire were not assessed, such as what specific nutritional information was provided, what type of vitamin tests were performed, and which nutrients participants would have liked more information about. Lastly, recall bias could have influenced self-reported experiences due to the retrospective nature of this study. This bias was limited by only including women who had given birth in the last two years.

## 5 Conclusion

This study showed that only a minority of pregnant women following a vegan diet received information and recommendations from their care providers concerning their diet. To improve healthcare for pregnant women following a vegan diet, obstetric healthcare providers should inquire about diet during the first prenatal consultation. Additionally, we recommend that women should be directed to reliable sources of dietary information that can be found in leaflets and on websites, or referred to a dietitian specialized in vegan diets.

Adequate access to nutritional information for pregnant women following a vegan diet is key, as optimal nutrition plays an important role in preventing maternal and fetal complications. Therefore, it is vital to ensure that pregnant women following a vegan diet receive the information and recommendations they need in order to be as healthy as possible during their pregnancy. In this way, they can optimize both their own health and the lifelong health of their children.

**Ethical disclosure:** This study was conducted according to the guidelines laid down in the Declaration of Helsinki. All procedures involving research study participants were approved by the Ethics Review Committee of the Faculty of Health Medicine and Life Sciences of Maastricht University as part of the license of the Master Health Education and Promotion program, registration code FHML/HEP_202.074. Written informed consent was obtained from all subjects.

## CRediT authorship contribution statement

**Deidre Meulenbroeks:** Writing – original draft, Methodology, Formal analysis, Data curation, Conceptualization. **Daisy Jonkers:** Writing – review & editing. **Hubertina Scheepers:** Writing – review & editing, Methodology, Conceptualization. **Jessica Gubbels:** Writing – review & editing, Supervision, Methodology, Conceptualization.

## Funding

This research did not receive any specific grant from funding agencies in the public, commercial, or not-for-profit sectors.

## Declaration of competing interest

The authors declare that they have no known competing financial interests or personal relationships that could have appeared to influence the work reported in this paper.

## Data Availability

Data will be made available on request.
