# Supplementary material for: Obstetric healthcare experiences and information needs of Dutch women in relation to their vegan diet during pregnancy
Source: Prev Med Rep. 2024 Oct 24;48:102916. doi: 10.1016/j.pmedr.2024.102916 (PMC11554913; doi:10.1016/j.pmedr.2024.102916)
Supplement: Supplementary Data 2 [file mmc2.docx]

**S2 – Questionnaire**

1. What is your date of birth?
2. What is your highest education?
   - primary school
   - secondary school
   - Intermediate vocational education
   - higher vocational education
   - university
3. When was the last time you gave birth?
   - 2017 or longer time ago -> end of the questionnaire
   - 2018, 2019, 2020
4. What was the date you gave birth the last time?
5. What was your due date?
6. How many times did you give birth before this pregnancy? (Gestational age >24 weeks)
7. Did you follow a vegan during the entire pregnancy, with less than once a month ingesting an animal product?
   - Yes
   - No -> end of questionnaire
8. Did you smoke during pregnancy?
9. What was your length and weight just before your last pregnancy?
10. Were there any complications during pregnancy?
    - No, there were no complications
    - Yes, gestational diabetes
    - Yes, high blood pressure
    - Yes, pre-eclampsia
    - Yes, the growth of the baby was too small
    - Yes, the growth of the baby was too high
    - Yes, other complications, namely ……………..
11. How did you give birth?
    - Vaginal birth
    - Vacuum extraction
    - Forceps delivery
    - Caesarean section
12. What was the birth weight of your baby? (gram)
13. Was your baby admitted to the hospital, and if so, why?
    Yes, because of preterm delivery
    Yes, because of neonatal infection
    Yes, because of low Apgar / asphyxia
    Yes, because of hypoglycemia
    Yes, because of hyperbilirubinemia
    Yes, because of low birth weight
    Yes, for observation of cardiac arrhythmia
    Yes, for observation after general anesthesia
    No
14. Did you start breastfeeding after delivery?
    - Yes
    - No -> go to question 18
15. How many weeks did you breastfeed your child?
16. Did you use any supplements during this time?
17. What supplements did you take?
    - Multivitamins
    - Vitamin D
    - Omega 3
    - Iron
    - Vitamin B12
    - Iodine
    - Other supplements, namely ………….
18. Did you have any specific questions or need for information about your vegan diet during pregnancy?
    - No -> go to question 20
    - Yes, about my nutritional intake during pregnancy
    - Yes, about supplements during pregnancy
    - Yes, about the effect of my diet on the growth of the baby
    - Yes, about the chance of maternal complications during pregnancy
    - Yes, about the change of fetal complications during pregnancy
    - Yes, about my nutritional intake during lactation
    - Yes, about supplements during lactation
    - Yes, other, namely ………..
19. When did you need this information?
    - Before I was pregnant
    - Between 4 and 12 weeks of my pregnancy
    - Between 12 and 28 weeks of my pregnancy
    - Between 28 weeks and the end of my pregnancy
    - Postpartum
20. Who was responsible for your check-ups during pregnancy?
    - The midwife
    - The gynecologist in the hospital
21. Did your midwife or gynecologist ask about your diet?
    - Yes
    - No, that’s why I told them myself
    - No, and I did not tell them myself -> go to question 29
    - I can’t remember
22. Did your obstetrical healthcare provider give you information about the possible effect of your diet on your pregnancy?
23. Did your obstetrical healthcare provider perform extra checks during pregnancy because of your diet?
    - Yes
    - No -> go to question 27
    - I can’t remember -> go to question 27
24. Did your obstetrical healthcare provider suggest these extra checks?
    - Yes
    - No, I asked for extra checks myself
25. What extra checks were performed?
    - Extra blood tests
    - Extra ultrasound(s) -> go to question 43
    - Other, namely ……………… -> go to question 43
26. What extra blood tests were performed?
    - Haemoglobin
    - Vitamins
    - I cannot remember
    - Other tests, namely ………….
27. Did your obstetric healthcare provider advise you to visit a dietitian during pregnancy because of your diet?
    - Yes, but I went to a dietitian already
    - Yes, that is why I went to a dietitian
    - Yes, but I decided not to go -> go to question 29
    - No -> go to question 29
    - No, but I did to a dietitian
28. Did you feel like your dietitian had enough knowledge of the vegan diet to give you proper advice about your diet during pregnancy?
29. Should your obstetric healthcare provider ask about your diet?
    - Yes
    - No, I do not think it is relevant information
    - No, someone with a different diet should tell this their selves
30. Who should provide information about the vegan diet in pregnancy?
    - The midwife or gynecologist
    - Dietitian
    - Nutritional Centre
    - Folder with reliable information
    - Website with reliable information
    - General practitioner
    - I prefer to look for information myself
    - I would not like to receive any extra information because I do not think this is necessary
    - From some else, namely ……………
31. Do you want to share other information about your diet during pregnancy?
    - No
    - Yes, namely ………….
