# Supplementary material for: Obstetric healthcare experiences and information needs of Dutch women in relation to their vegan diet during pregnancy
Source: Prev Med Rep. 2024 Oct 24;48:102916. doi: 10.1016/j.pmedr.2024.102916 (PMC11554913; doi:10.1016/j.pmedr.2024.102916)
Supplement: Supplementary Data 3 [file mmc3.docx]

**Appendix A.**

Maternal and fetal outcomes of pregnant women on a vegan diet (n=193)

| **Outcomes** | ***n (%) ^a^*** |
| --- | --- |
| **Gestation at delivery** Preterm (32+0 – 36+6 weeks) Full term (37+0 – 40+6 weeks) Imminent post term (41+1 – 42+ weeks) | 15 (7.8) 126 (65.3) 52 (26.9) |
| **Complications during pregnancy** Gestational diabetes Pregnancy induced hypertension Pre-eclampsia Foetal growth restriction Macrosomia Placental abruption | 7 (3.6) 7 (3.6) 5 (2.6) 6 (3.1) 6 (3.1) 1 (0.5) |
| **Mode of birth** Vaginal birth Assisted vacuum extraction Caesarean section | 151 (78.2) 14 (7.3) 28 (14.5) |
| **Birth weight** Small for gestational age (<10th percentile) Normal (10th – 90th percentile) Large for gestational age>90 percentile | 22 (11.4) 158 (81.9) 12 (6.8) |

N deviates from total sample size due to missing values

^a^ *n* (%) =number of participants (percentage)
